# Characteristics and reference ranges of CD4^+^T cell subpopulations among healthy adult Han Chinese in Shanxi Province, North China

**DOI:** 10.1186/s12865-020-00374-9

**Published:** 2020-08-03

**Authors:** Hong-Qing Niu, Xiang-Cong Zhao, Wei Li, Jian-Fang Xie, Xiao-Qing Liu, Jing Luo, Wen-Peng Zhao, Xiao-Feng Li

**Affiliations:** Department of Rheumatology, the Second Hospital of Shanxi Medical University, 382 Wuyi Road, Taiyuan, 030001 China

**Keywords:** CD4^+^T cell subpopulations, Regulatory T cells, Reference values, Flow cytometry, Adults

## Abstract

**Background:**

Immunophenotyping of blood lymphocytes is an essential tool to evaluate the immune function of patients with immunodeficiency or autoimmunity. Predominately identified CD4^+^T cell subsets, Th1, Th2, Th17, as well as regulatory T (Treg) cells, play crucial roles in several immunological and pathological conditions. Considering the variations in cell counts among populations and ethnicities, specific CD4^+^T cell subset reference values need to be locally established to enable meaningful comparisons and accurate data interpretation in clinical and research settings. Therefore, the aim of this study was to establish distributions and reference ranges for blood CD4^+^T cell subpopulations in age- and sex-balanced healthy adults of a Han Chinese population in Shanxi Province, North China.

**Methods:**

Peripheral blood CD4^+^T cell subsets were examined in 150 healthy volunteers (75 males, 75 females) aged 20–70 years with a four-color FACSCalibur flow cytometer.

**Results:**

Reference value percentages (absolute counts, cells/μl) were defined as 95% of the population for cell types as follows: CD4^+^T, 23.78–51.07 (360–1127); Th1, 0.43–39.62 (2.64–276.21); Th2, 0.27–3.57 (1.80–27.14); Th17, 0.22–2.62 (1.10–19.54); and Treg, 2.17–7.94 (13.47–64.58). The ranges for the Th1:Th2 and Th17:Treg ratios were 0.59–52.37 and 0.04–0.76, respectively. Notably, a significant increase was observed in the values of Treg cells in older individuals, and the numbers of Treg cells in females also tended to decrease when compared to those in males. Therefore, we established the distribution and reference range of CD4^+^T cell subsets based on age and sex, demonstrating the lowest values of Treg cells in younger females.

**Conclusions:**

Collectively, our data provide population-, age-, and sex-specific distributions and reference ranges of circulating CD4^+^T cell subpopulations, which can be adopted to guide clinical decisions and interpretation of immunophenotyping data in the Han Chinese population in Taiyuan, Shanxi Province, China. In addition, the low expression of peripheral Treg cells in younger females may be associated with the predisposition of females to autoimmune diseases.

## Background

Immunophenotyping of peripheral blood lymphocytes with monoclonal antibodies via flow cytometry has proven to be a useful tool to evaluate the immunological function of patients with immunodeficiency, autoimmunity, transplantation, tumor, or infection, and is also valuable to monitor treatment responses and disease progression [1–4]. Currently, flow cytometry is the most precise and reliable tool to assess the immunological status, and technical advances in this field have provided new opportunities to better determine the function and improve cell phenotyping of peripheral lymphocytes.

As the predominant lymphocyte subset, CD4^+^T cells play crucial roles in numerous conditions such as infection, autoimmunity, transplantation, and tumor. Upon antigenic stimulation, CD4^+^T cells adopt one of two opposing fates: a helper T (Th) cell specialized in supporting the clearance of infections or a regulatory T (Treg) cell that functions to attenuate immune responses [5]. Th1 cells play a critical role in host defense against intracellular pathogens and in autoimmune diseases by producing a key inflammatory cytokine interferon (IFN)-γ [6]. Th2 cells are important in humoral immunity and protection from helminth infection and are central to the pathogenesis of several allergic inflammatory diseases [7]. Th17 cells provide critical support for immunity against extracellular bacteria and fungi and are the leading actors in autoimmunity [5], whereas Treg cells suppress the autoreactive activities of effector CD4^+^T cells and thus maintain immune tolerance [8].

Currently, reference values of lymphocyte subsets are utilized to increase the accuracy of data interpretation in clinical and research settings. Region-specific reference ranges for adult peripheral blood T cells, B cells, and NK cells have also been determined in certain countries [9–16]. However, other than one study from Italy in 2016 [17], no other study has investigated the characteristics and distributions of circulating CD4^+^T cell subpopulations in a wide range of healthy subjects from the Han Chinese population. To fill this knowledge gap, we determined the percentages and absolute counts of peripheral blood CD4^+^T cell subpopulations in an age- and sex-balanced population of healthy adults of Han Chinese ethnicity to establish monocentric reference values and also analyzed the characteristics of CD4^+^T cell subsets based on sex and age.

## Results

### General characteristics and reference values

**Fig. 1:**
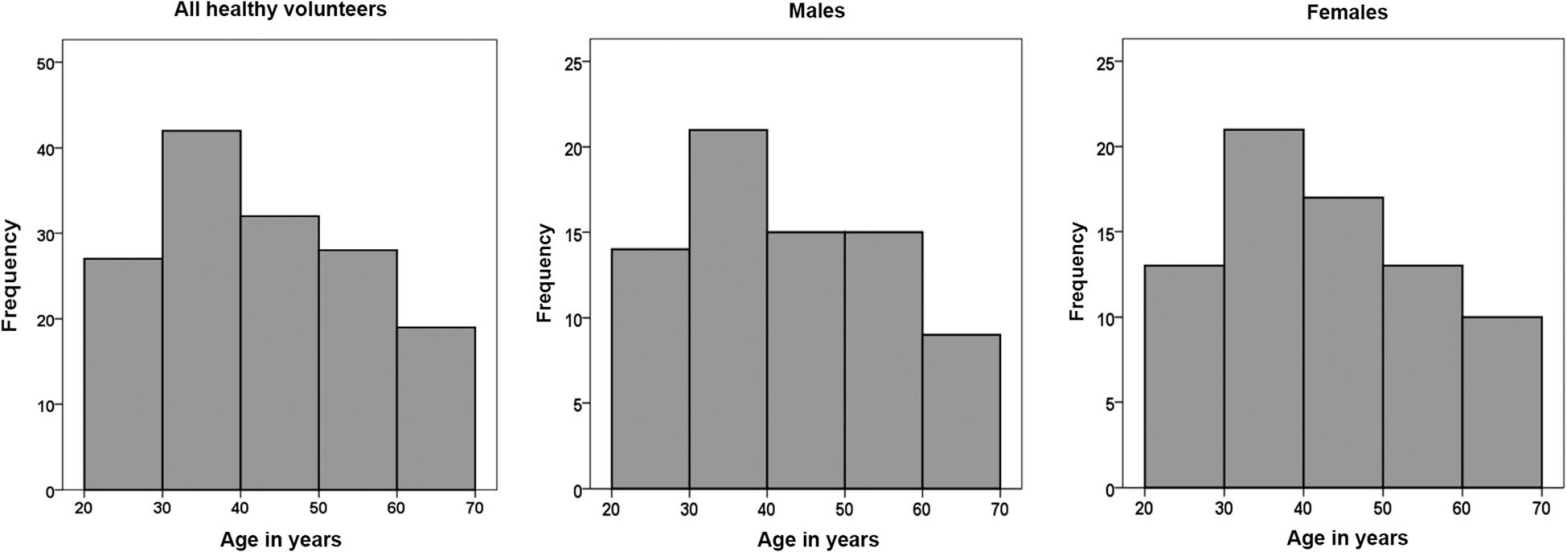
Age distribution among all healthy volunteers, males or females. The age distributions in the present study were nearly identical across all groups

Among the 150 healthy volunteers, 75 were males and 75 were females; their ages ranged from 20 to 70 years (median age, 43 years). The age distributions in the present study were nearly identical for all healthy volunteers and for male and female subjects (Fig. 1).

Table 1 shows the mean, median, interquartile ranges, and reference ranges for the Th1 cells (CD4^+^IFN-γ^+^), Th2 cells (CD4^+^IL-4^+^), Th17 cells (CD4^+^IL-17^+^), and Treg cells (CD4^+^CD25^+^Foxp3^+^) as well as the ratios of Th1:Th2 and Th17:Treg cells. In addition, the percentages and absolute counts of lymphocyte subsets are summarized in Additional file 1: Table S1.

**Table 1:** Reference ranges of peripheral blood CD4^+^T subpopulations in 150 healthy adults and comparisons with previously published data

Parameters	Present study				Italy study [17]
	Mean	Median	Interquartile range	Reference ranges ^***a)***^	Reference ranges ^***a)***^
**CD4**^**+**^**T subpopulations (%)**					
Th1	16.97	16.39	13.49	0.43–39.62	6.66–8.01
Th2	1.35	1.19	0.97	0.27–3.57	4.86–5.98
Th17	1.14	1.07	0.77	0.22–2.62	9.50–11.47
Treg	4.80	4.74	2.17	2.17–7.94	0.59–0.79
Th1:Th2	16.96	13.40	15.14	0.59–52.37	1.33–1.54
Th17:Treg	0.26	0.21	0.20	0.04–0.76	0.06–0.11^*b****)***^
**CD4**^**+**^**T subpopulations (cells/μl)**					
Th1	116.87	105.77	103.75	2.64–276.21	144.20–171.40
Th2	9.32	8.25	7.42	1.80–27.14	106.40–129.90
Th17	7.75	7.02	5.98	1.10–19.54	201.30–242.00
Treg	32.73	30.58	18.82	13.47–64.58	12.79–17.41

Th1, Th2, Th17, and Treg cells (percentages) were gated from CD4^+^T cells. The absolute counts for each CD4^+^T subpopulation were calculated by multiplying the specific subset percentage by absolute CD3^+^CD4^+^T cell counts

^*a)*^ Reference ranges are defined as 95% of the population

^*b)*^ Treg:Th17 ratio

Overall, more CD4^+^T cells were observed compared to CD8^+^T cells in all volunteers who participated in the present study, and the median (25th and 75th percentiles) CD4:CD8 ratio was 1.36 (1.01–1.78). Of the CD4^+^T cell subpopulations, the numbers of Th1 cells were obviously higher than those of Th2, Th17, or Treg cells; the Th17 cell numbers were the lowest in the peripheral blood. The median (25th and 75th percentiles) Th1:Th2 and Th17:Treg ratios were 13.40 (6.98–22.12) and 0.21 (0.14–0.34), respectively.

### Comparisons of reference ranges with published data

To the best of our knowledge, there was only one previous study from Northeast Italy [17] that determined the reference ranges of Th1, Th2, Th17, and Treg cells. Thus, our data were compared with values reported in that study. The reference ranges of CD4^+^T cell subpopulations obtained in our study were considerably different from those reported in the previous study (Table 1). For example, the percentages and absolute counts of Th2 and Th17 cells were lower and the numbers of Treg cells were higher in the present study than in the Northeast Italy study. In addition, the reference ranges of Th1 cells, the Th1:Th2 ratio, and the Th17:Treg ratio were larger in our study than in the Italy study.

Furthermore, the values of lymphocyte subsets were also compared with those reported from subjects of other parts of China or from different ethnic groups. As shown in Additional file 1: Table S2, our values of lymphocyte subsets clearly differed from those of populations determined in other parts of China. The T cell percentages, CD8^+^T cell percentages and absolute counts obtained in the present study were higher than those in a previous study from Beijing, China, whereas the CD4:CD8 ratio was lower in our population. Although the ranges of T cells and CD8^+^T cells and the CD4:CD8 ratio in our study were comparable to the reported values based on a larger cohort from Hong Kong, the percentage and absolute count values of CD4^+^ T and B cells in our population were lower than those reported for the Hong Kong population, and the values of NK cells were higher in our study.

The reference values of CD4^+^T cell proportions and absolute counts were similar to those of other Asian populations but were lower than those of German and American populations. The absolute counts and percentage reference values of CD8^+^T cells in North Chinese individuals were higher than those of Koreans and Germans; however, the CD8^+^T absolute count ranges were lower than those of Americans. In addition, the CD4:CD8 ratio differed from the Korean and German data. Furthermore, the reference ranges of our study were different from the reported Omani ranges (Additional file 1:Table S2).

### Age-specific characteristics

**Fig. 2:**
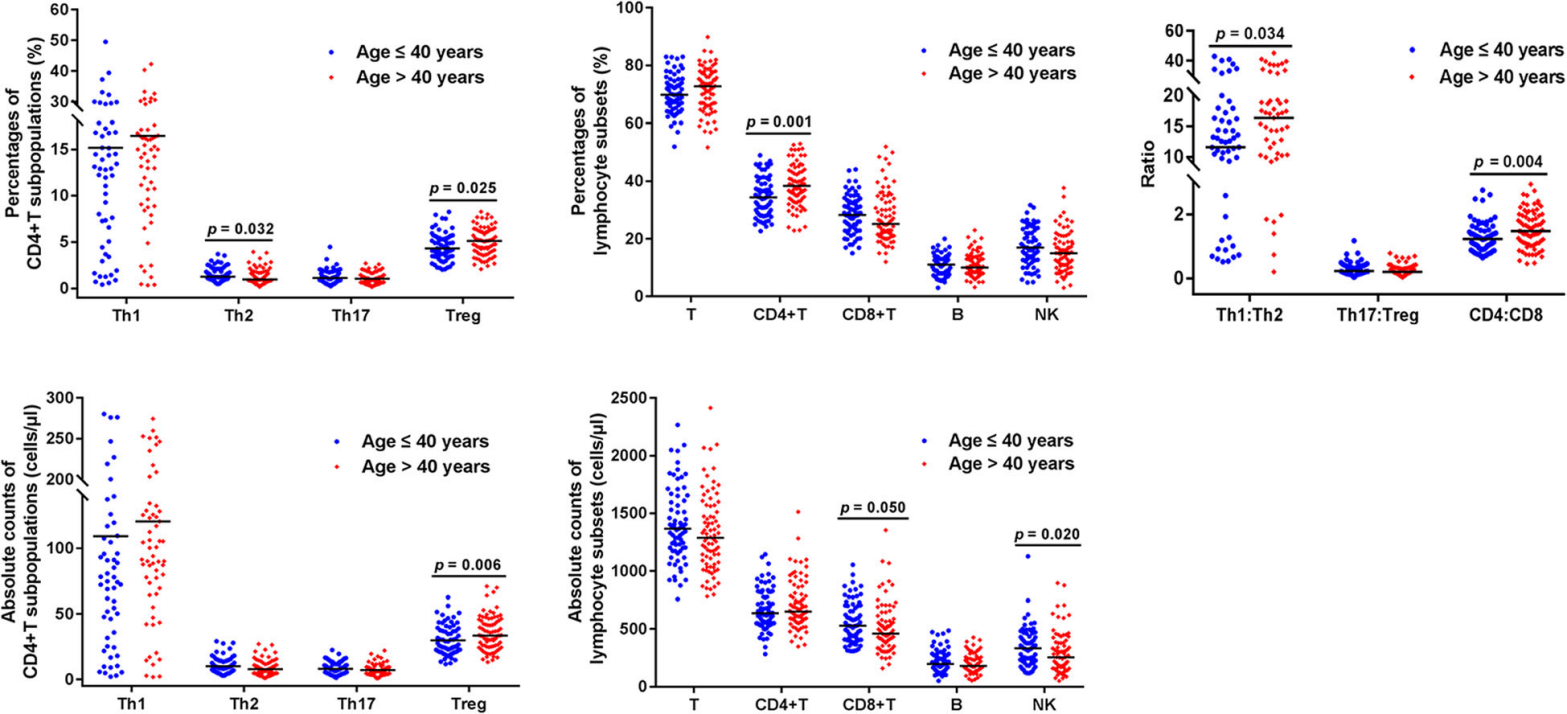
Comparison of CD4^+^T cell subpopulations and lymphocyte subsets between populations aged ≤40 years and >  40 years. Flow-cytometric enumerations of T cells, B cells, NK cells, and CD4^+^T cell subpopulations were performed. The differences in the numbers of each lymphocyte subset and ratios of Th1:Th2, Th17:Treg, and CD4:CD8 were compared. Black bars: medians. *P*-values were determined with the Wilcoxon rank-sum test

To analyze the influence of age on the distribution and reference values of CD4^+^T cell subpopulations, the population was divided into two groups as follows: ≤ 40 years (*n* = 71) and >  40 years (*n* = 79). The results showed that the numbers of Treg cells significantly differed between the age groups. The median percentages and absolute counts of Treg cells were 4.31% and 25.82 cells/μl in the younger group; these values were significantly lower than those in older subjects (5.11% and 33.48 cells/μl respectively; *Z* = − 2.245, *p* = 0.025; *Z* = − 2.731, *p* = 0.006). Moreover, the median percentages of Th2 cells in the younger group were higher than those in the older subjects (1.25% vs. 0.97%, *Z* = − 2.146, *p* = 0.032). The median numbers of Th1 cells in younger subjects were lower than those in subjects beyond the age of 40 years, but the differences were not statistically significant. No significant differences were observed for the Th17 cells. Furthermore, the median Th1:Th2 ratio in the younger subjects was lower (11.57 vs. 16.32, *Z* = − 2.115, *p* = 0.034) than that in older subjects. Although the Th17:Treg ratio in the younger subjects was higher than that in the older ones (0.23 vs. 0.19), the difference was not significant (*Z* = − 1.918, *p* = 0.055; Fig. 2).

Furthermore, we analyzed age-related differences in the distribution of lymphocyte subsets. As shown in Fig. 2, the median percentage of CD4^+^T cells in healthy subjects ≤40 years (34.40%) was significantly lower than that in the older group (38.35%; *Z* = − 3.333, *p =* 0.001). In contrast, the absolute counts of NK cells in the younger subjects were higher than those in the older individuals. Moreover, the median CD4:CD8 ratio was significantly lower in the younger group (1.23 vs. 1.47, *Z* = − 2.887, *p =* 0.004).

**Fig. 3:**
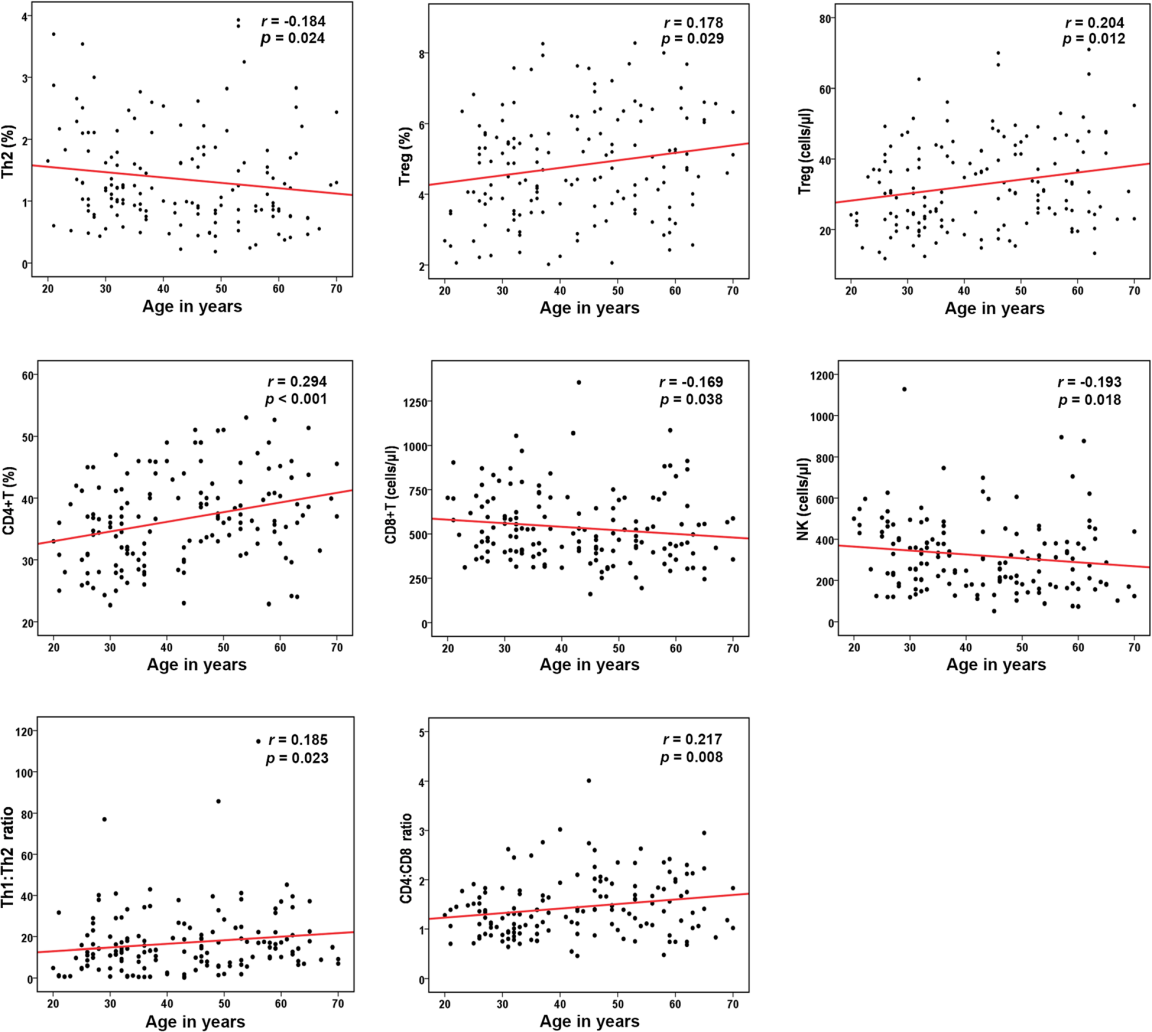
Scatter diagram of the correlations between lymphocyte subset numbers and age and linear trends. Spearman correlation analysis showed statistical significance between age and Th2 proportions, Treg proportions and counts, CD4^+^T proportions, CD8^+^T counts, NK cell counts, the Th1:Th2 ratio, or the CD4^+^:CD8^+^ ratio

In addition, we analyzed the correlation between age and various immune cell subtypes. A weak positive correlation was observed between age and Treg proportions and counts, CD4^+^ proportions, the Th1:Th2 ratio, and the CD4^+^:CD8^+^ ratio. However, correlations between age and Th2 proportions, CD8^+^ counts, and NK cell counts were significantly weak and negative (Fig. 3).

### Sex-specific characteristics

**Fig. 4:**
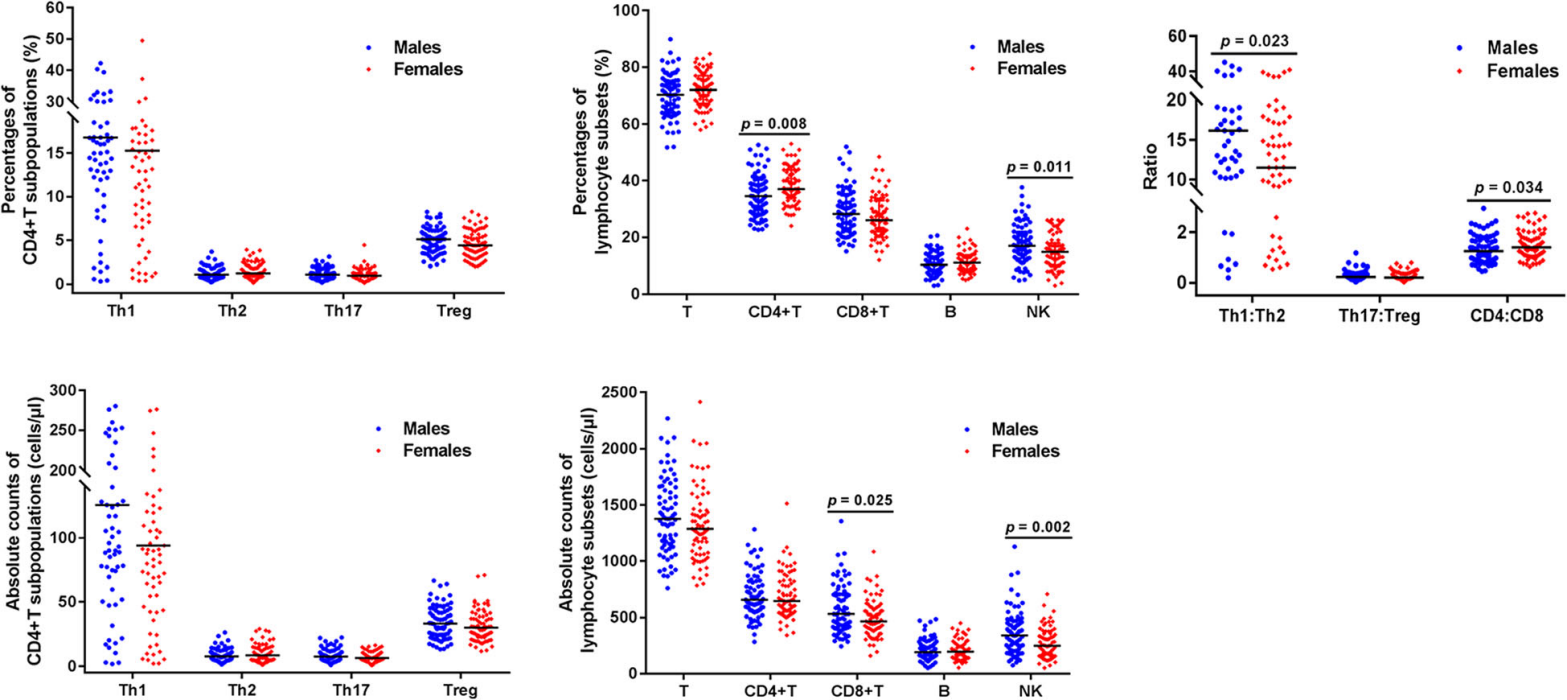
Comparison of CD4^+^T cell subpopulations and lymphocyte subsets between males and females. Flow-cytometric enumerations of T cells, B cells, NK cells, and CD4^+^T cell subpopulations were performed, and the numbers of each lymphocyte subset of males were compared to those of females. Black bars: medians. *P*-values were determined with the Wilcoxon rank-sum test

To analyze any sex-related differences, the 150 healthy adults were divided into two groups, males (*n* = 75) and females (*n* = 75). Unlike age-related differences, there were no significant sex-related differences in the distribution of CD4^+^T cell subpopulations. The median percentages and absolute counts of Th1 and Treg cells in the female group were lower than those in the male group, but the differences were not statistically significant. However, the ratio of Th1:Th2 cells in male subjects was higher than that in females (median: 16.17 vs. 11.51; *Z* = − 2.268, *p* = 0.023). There was no significant difference in the ratio of Th17:Treg cells between males and females (median: 0.23 vs. 0.20; *Z* = − 0.355, *p* = 0.722; Fig. 4).

We also demonstrated the differences between sex-grouped populations in terms of the percentages and absolute counts of NK cells (*Z* = − 2.555, *p* = 0.011; *Z* = − 3.116, *p* = 0.002). The percentages of CD4^+^T cells were significantly decreased in male subjects compared to those in females (median: 34.56% vs. 37.00%; *Z* = − 2.637, *p* = 0.008), and the absolute counts of CD8^+^T cells were significantly increased in male subjects compared to those in female subjects (median: 529 cells/μl vs. 466 cells/μl; *Z* = − 2.236, *p* = 0.025). Moreover, the median CD4:CD8 ratio in male subjects was lower than that in female subjects (median: 1.25 vs. 1.40; *Z* = − 2.114, *p* = 0.034; Fig. 4).

Additionally, we compared the difference in various immune cells between pre-menopausal females (*n* = 50) and post-menopausal females (*n* = 25). The results showed that the proportions of Th17 cells and the Th17:Treg ratio were higher in pre-menopausal females than in post-menopausal females [1.14 (0.70, 1.43)% vs. 0.74 (0.51, 1.12)%, 0.22 (0.16, 0.36) vs. 0.16 (0.11, 0.26), respectively; *Z* = − 2.096, *p* = 0.036; *Z* = − 2.332, *p* = 0.020]. The distribution of other immune cells showed no difference in the two groups.

### Distribution and reference values in populations grouped by age and sex

The results summarized clearly indicated that age and sex influence the distribution of various immune cell subsets. Therefore, the age- and sex-specific reference values of lymphocyte subpopulations for the healthy Han Chinese population in Taiyuan, Shanxi Province, North China, were determined. Herein, the 150 healthy adults were divided into four groups as follows: male and ≤ 40 years (*n* = 36); female and ≤ 40 years (*n* = 35); male and >  40 years (*n* = 39); and female and >  40 years (*n* = 40). The median and reference ranges for CD4^+^T cell subpopulations are shown in Table 2.

**Table 2:** Distribution and reference ranges of CD4^+^T cell subpopulations based on age and sex

Parameters	Age (years)	Median		***P***-value	Reference ranges ^***a)***^	
		Male	Female		Male	Female
**CD4**^**+**^**T subpopulations (%)**						
Th1	≤ 40	16.77	13.43	0.191	0.58–39.41	0.40–49.52
	> 40	17.10	16.19		0.33–42.26	0.41–31.06
Th2	≤ 40	1.19	1.26	0.083	0.43–3.70	0.52–3.54
	> 40	0.99	0.94		0.24–2.83	0.18–3.92
Th17	≤ 40	1.08	1.15	0.229	0.20–3.14	0.23–4.46
	> 40	1.12	0.91		0.31–2.70	0.16–2.05
Treg	≤ 40	4.92	3.88	0.043 ^***b)***^	2.06–8.26	2.02–7.93
	> 40	5.14	4.74		2.56–8.00	2.06–8.26
Th1:Th2	≤ 40	13.06	9.89	0.015 ^***c)***^	0.51–77.00	0.53–40.91
	> 40	17.43	14.39		0.20–114.76	1.40–84.62
Th17:Treg	≤ 40	0.22	0.25	0.059	0.04–1.18	0.03–0.76
	> 40	0.23	0.17		0.05–0.70	0.03–0.78
**CD4**^**+**^**T subpopulations (cells/μl)**						
Th1	≤ 40	106.02	80.64	0.125	2.90–280.46	2.11–311.24
	> 40	128.70	104.85		1.82–260.03	2.37–273.85
Th2	≤ 40	8.14	9.29	0.341	2.94–23.57	2.90–29.17
	> 40	7.56	8.17		1.41–26.30	1.10–26.93
Th17	≤ 40	7.33	6.48	0.423	0.91–22.44	1.26–16.34
	> 40	8.27	6.39		1.56–22.09	0.82–15.28
Treg	≤ 40	29.68	24.64	0.022 ^***b)***^	13.54–62.54	11.76–50.83
	> 40	36.20	31.20		13.26–66.61	14.78–70.93

^***a)***^ Reference ranges are defined as 95% of the population

^***b)***^ Numbers of cell subtypes with significant differences among members of age- and sex-grouped populations

^***c)***^ ratio of cell subtypes with significant differences among members of age- and sex-grouped populations

Statistics: Kruskal-Wallis χ^2^ test

We also compared the distributions of cell subtypes in the four age- and sex-grouped populations. The proportions and absolute counts of Treg cells and the Th1:Th2 ratio were significantly different among the four groups, with the highest percentages and absolute counts of Treg cells in the group of older males and the lowest values in younger females (Table 2). In addition, the differences in CD4^+^T and NK proportions, CD8^+^T and NK counts, and the CD4:CD8 ratio were significant among the groups, with the highest NK percentages and counts in the group of younger males and the lowest values in older females (Additional file 1: Table S3).

## Discussion

Clinical diagnosis and research outcomes become more meaningful and accurate when reliable reference ranges of lymphocyte subtypes are available for comparisons with respect to the local population. Flow cytometry is currently considered the “gold standard” for the identification of lymphocyte subsets and evaluation of immune function [18]. In the present study, we analyzed the distributions and established the reference ranges of lymphocyte subsets and extended our datasets of CD4^+^T subpopulations using flow cytometry based on a healthy Han Chinese population in Taiyuan, Shanxi Province, North China.

Although various methodologies and gating strategies have been established to define lymphocyte subsets, the CD45 gating strategy is considered the most appropriate approach to accurately and reliably identify lymphocyte populations [14]. Moreover, distribution and reference ranges of circulating Th1, Th2, Th17, and Treg cells were assessed using a CD4 gating strategy. Immunofluorescence staining of specific surface markers in combination with intracellular transcription factor and/or cytokine staining was used to identify CD4^+^T cell subsets [18, 19]. These CD4^+^T cells were further labeled with functional intracellular molecules (IFN-γ for Th1, IL-4 for Th2, IL-17 for Th17, and Foxp3 for Tregs) or specific extracellular markers (CD25 for Tregs).

To the best of our knowledge, only one previous study from Northeast Italy [17] reported the reference ranges of Th1, Th2, Th17, and Treg cells. Obvious differences in the reference values of CD4^+^T cell subpopulations were observed between the present study and the published data from Northeast Italy [17], which could be associated with race and sample sizes. Of note, among the primarily identified CD4^+^T cell subsets, Th1, Th2, Th17, and Treg cells were associated with different tagging strategies. The Italy study adopted chemokine receptors and other surface molecules for CD4^+^T cell immunophenotyping; for example, CD195 (CCR5) was used to label Th1 cells, whereas CD194 (CCR4) and CD161 were used to mark Th2 and Th17 cells, respectively. Therefore, the immunofluorescence staining of Th1, Th2, and Th17 cells in the Italian study differed from that adopted in our study, which could also contribute to the observed differences in reference ranges. Treg cells are an immunosuppressive subset of CD4^+^T cells characterized by expression of the master transcription factor Foxp3 [20]. However, similar to the present study, Treg cells were stained with anti-CD4 FITC, anti-CD25 PE, and anti-Foxp3 PerCP in the Italian study [17], and yet different values were obtained. One study from Philadelphia, USA, reported the overall frequency of the polyclonal Treg cell population to be approximately 5–15% of total CD4^+^T cells [20], which is higher than that obtained in the present study. This difference could also result from different racial factors and experimental conditions.

We further demonstrated clear differences in the values of CD4^+^T cell subpopulations according to the age of the participants, with higher values for Treg cells (both percentages and absolute counts) in the older group (> 40 years old). In addition, Togashi et al. [21] reported that the abundance of Treg cells in the peripheral blood tends to increase with aging, which supports our data. Moreover, a significant positive correlation was observed between age and Treg cell numbers (both percentages and absolute counts). Sex also has an influence on the lymphocyte subset distribution [13, 14]. Indeed, we found lower values of Th1 and Treg cells in females than in males, but the differences were not significant. Moreover, a higher percentage of CD4^+^T cells in females was also observed. These differences are likely related to different hormonal effects [14] and confirm the influence of sex on peripheral lymphocyte subsets.

These significant differences above clarify the impact of age and sex on the distribution of certain lymphocyte subpopulations. Thus, the distribution and reference ranges of lymphocyte subsets in healthy participants were evaluated based on age and sex groupings, demonstrating the highest percentages and absolute counts of Treg cells in the group of older males and the lowest values in younger females.

Treg cells are an immunosuppressive subset of CD4^+^T cells and play pivotal roles in the prevention of autoimmunity and maintaining immune tolerance. Deficiency in either the number or the function of Treg cells may lead to the breakdown of immune homeostasis and development of autoimmune diseases [22]. Most autoimmune diseases, such as systemic lupus erythematosus and rheumatoid arthritis, have a strong female predilection and preferably occur in young women [23]. In the present study, Treg cells displayed age- and sex-biased expression, with younger females showing a lower expression than older males, a finding that may explain the susceptibility of young females to autoimmune diseases. Further studies are required to explore the exact underlying mechanism.

Indeed, differences in other lymphocyte subsets, including B cells and NK cells, have also been confirmed in the present study via comparisons of reference values obtained from populations of different parts of the world. These inconsistencies could be attributed to technical advances in flow cytometry, different sample sizes, ethnic variations, and other factors affecting the immune function of individuals. B cells discriminate pathogens from self, eliminate infections, ‘encode’ a memory of pathogen encounters and provide life-long immunity, or produce auto-antibodies to induce autoimmune response [24]; NK cells play important roles in the innate immune response against tumors [25]. Furthermore, our study demonstrated lower NK cell values in older subjects, which is consistent with the findings of a prior study [14]. This phenomenon may be associated with the decreased function of immune surveillance in the older population. However, the sex-wise distribution involved in the number of peripheral NK cells observed in the present study remains to be confirmed and investigated in future studies.

## Conclusions

In summary, we analyzed the characteristics and established local reference values for a wide range of peripheral blood CD4^+^T cell subpopulations from healthy Han Chinese adults in Taiyuan, Shanxi Province, North China. This is the first study to investigate and compare reference ranges based on both the absolute number and percentage of CD4^+^T cell subpopulations in an age- and sex-balanced population within China. Age, sex, and ethnicity emerged as major factors contributing to the variations in lymphocyte phenotype composition. The significantly lower expression of peripheral Treg cells in younger females may be associated with the predisposition of females to autoimmune diseases. Accordingly, it is recommended that each laboratory determine its local reference ranges to facilitate the interpretation of research results and clinical decision-making. Of note, the main limitation of our study is the sample size when considering reference values based on age- and sex-grouped individuals. Therefore, these findings should be evaluated further, and reference ranges need to be established over a wider scale.

## Methods

### Subjects and sample collection

A total of 150 adults (75 males, 75 females) ranging from 20 to 70 years of age (median age, 43 years) were recruited for the present study from May to September of 2018. All participants were healthy volunteers from the City of Taiyuan, Shanxi Province, China, without evidence of inflammatory syndromes, autoimmune or inflammatory diseases, or recent acute or chronic infectious diseases, and no history of cancer. None of the subjects had recently received steroids or immunosuppressive drugs or other medications known to affect the immune system. There was 100% homogeneity for all volunteers with respect to the Han Chinese ethnicity. Whole-blood samples (2 ml) from all subjects were collected into ethylenediaminetetraacetic acid (EDTA) anticoagulant vacutainer tubes and were stained and analyzed within 6 h of collection for flow-cytometric analysis.

### Cell preparation, staining, and flow cytometry

Peripheral blood lymphocyte phenotypes and CD4^+^T cell subpopulations were determined using a FACSCalibur flow cytometer (two lasers, four colors; BD Biosciences, San Jose, CA, USA) with fluorochrome-labeled monoclonal antibodies. For immunofluorescence staining of circulating lymphocyte subsets, two sets of four-color monoclonal antibody combinations were used. Twenty microliters of anti-CD45-PerCP/anti-CD3-FITC/anti-CD4-APC/anti-CD8-PE reagent was pipetted into the bottom of a Trucount tube, labeled A, and 20 μl of anti-CD45-PerCP/anti-CD3-FITC/anti-CD19-APC/anti-CD16 + 56-PE reagent was pipetted into the bottom of a Trucount tube, labeled B. Thereafter, 50 μl of the anticoagulated blood samples was added to tubes A and B, respectively. The tubes were capped and vortexed gently. After incubation at room temperature (20–25 °C) for 15 min in the dark, 450 μl of 1X FACS lysing solution was added to each tube. The tubes were further incubated for 15 min in the dark at room temperature (20–25 °C) and then analyzed using the flow cytometer. For each sample, a minimum of 10,000 events was acquired and analyzed by Cell Quest software. Post-acquisition of compensation was performed before sample analysis. Lymphocytes were identified by their strong CD45 expression and low side scatter. The percentages and absolute counts of CD3^+^T cells, CD3^+^CD4^+^T cells, CD3^+^CD8^+^T cells, CD3^−^CD19^+^B cells, and CD3^−^CD16^+^CD56^+^NK cells were automatically calculated using BD Multitest software (BD Biosciences). Gating strategies are described in Additional file 2: Figure S1.

Three sets of multi-color monoclonal antibody combinations were used for immunofluorescence staining of circulating CD4^+^T cell subpopulations. For the analysis of Th1, Th2, and Th17 cells, 80 μl of anticoagulated blood was stimulated with 10 μl phorbol 12-myristate 13-acetate (PMA), 10 μl ionomycin, and 1 μl GolgiStop; following this, incubation was carried out for 5 h at 37 °C. The blood samples were divided into tubes A and B, followed by staining with anti-CD4-FITC at room temperature (20–25 °C) in the dark for 30 min. The cells were fixed and permeabilized using Cytofix/Cytoperm reagents in a 4 °C incubator for 30 min. The samples were then stained with anti-IFN-γ-APC and anti-IL-17A-PE in tube A, and with anti-IL-4-PE in tube B at room temperature (20–25 °C) in the dark for 30 min. The cells were washed with PBS and analyzed by flow cytometry. For the analysis of Treg cells, 80 μl of anticoagulated blood was stained with anti-CD4-FITC and anti-CD25-APC at room temperature (20–25 °C) in the dark for 30 min and then fixed and permeabilized in a 4 °C incubator with 1 ml Cytofix/Cytoperm reagents, followed by staining with anti-Foxp3-PE for 30 min. The cells were washed and analyzed using the flow cytometer. Analysis of CD4^+^T subpopulations was performed using a fluorescence gating strategy based on CD4^+^/SSC log-gating. For immunophenotyping, CD4^+^T cells were defined as Th1 (CD4^+^IFN-γ^+^), Th2 (CD4^+^IL-4^+^), Th17 (CD4^+^IL-17^+^), and Tregs (CD4^+^CD25^+^Foxp3^+^) (see Additional file 2: Figure S2). A total of 10,000 cells per gate were detected and analyzed by Cell Quest software to acquire the frequencies of CD4^+^T subpopulations. Post-acquisition of compensation was performed by Cell Quest before sample analysis. Absolute counts of CD4^+^T subpopulations were calculated using the percentage of CD4^+^T cell subsets and the absolute counts of CD3^+^CD4^+^T cells. All immunofluorescence antibodies were purchased from BD Biosciences, and the results are expressed as percentages (%) of the parental lineage gate and absolute counts (cells/μl).

### Quality control

Internal quality-control procedures were carried out to assess instrument parameters and ensure accurate and reproducible enumeration. Daily calibration of the flow cytometer was performed using Calibrite 3 Beads and APC Beads (BD Calibrite™) for optical laser alignment and optimal hydrodynamic focusing settings, respectively. An external quality control procedure was also completed through participation in a performance assessment conducted by the Clinical Inspection Center of the National Health Commission in China.

### Statistical analysis

Data were analyzed with SPSS 20.0 statistical software (SPSS Inc., Cary, NC, USA). Medians and reference ranges were calculated for each lymphocyte phenotype. Reference ranges were defined as 95% of the population (from 2.5 to 97.5%). Continuous variables with non-normal distributions are expressed as the median and 25th and 75th percentiles (interquartile range). The influence of sex and/or age on the distribution of peripheral blood lymphocytes and CD4^+^T cell subsets was evaluated using the non-parametric Wilcoxon rank-sum test or Kruskal-Wallis χ^2^ test, as appropriate. Spearman correlation coefficients were used for correlation analysis. A *P*-value < 0.05 (two-sided) was considered statistically significant.

## Supplementary information

**Additional file 1 Table S1.** Reference ranges of peripheral blood lymphocyte subsets in 150 healthy adults. **Table S2.** Comparison of peripheral blood lymphocyte subset reference ranges from the present study with those published previously. **Table S3.** Distribution and reference ranges of lymphocyte subsets based on age and sex.

**Additional file 2 Figure S1.** Analysis of lymphocyte subsets by flow cytometry. A: Gating strategy for T cells, CD4^+^T cells, CD8^+^T cells: a expressed as a single parameter of CD45 and scatter; b T cells (CD3^+^); c CD4^+^T cells (CD3^+^CD4^+^); d CD8^+^T cells (CD3^+^CD8^+^). B: Gating strategy for B cells and NK cells: a expressed as a single parameter of CD45 and scatter; b expressed as a single parameter of CD3^−^ and scatter; c: B cells (CD3^−^CD19^+^); d: NK cells (CD3^−^CD16^+^CD56^+^). **Figure S2.** Analysis of CD4^+^T subpopulations by flow cytometry. A: Gating strategy for Th1 and Th17 cells: a expressed as a single parameter of CD4 and scatter; b Th1 (CD4^+^IFN-γ^+^); c Th17 (CD4^+^IL-17^+^). B: Gating strategy for Th2 cells: a expressed as a single parameter of CD4 and scatter; b Th2 (CD4^+^IL-4^+^). C: Gating strategy for Treg cells: a expressed as a single parameter of CD4 and scatter; b Treg (CD4^+^CD25^+^Foxp3^+^).

## Data Availability

The data and materials in the present study are available from the corresponding author on reasonable request.

## Abbreviations

APC: Allophycocyanin
CCR: C-C chemokine receptor
CD: Cluster of differentiation
EDTA: Ethylenediaminetetraacetic acid
FITC: Fluorescein isothiocyanate
Foxp3: Forkhead box p3
IFN-γ: Interferon-γ
IL: Interleukin
NK: Natural killer cells
PerCP: Peridinin chlorophyll protein
SSC: Side scatter
Th: Helper T cells
Treg: Regulatory T cells
