# Supplementary material for: Characteristics and reference ranges of CD4+T cell subpopulations among healthy adult Han Chinese in Shanxi Province, North China
Source: BMC Immunol. 2020 Aug 3;21:44. doi: 10.1186/s12865-020-00374-9 (PMC7397677; doi:10.1186/s12865-020-00374-9)
Supplement: Supplementary file 1 — Additional file 1 Table S1. Reference ranges of peripheral blood lymphocyte subsets in 150 healthy adults. Table S2. Comparison of peripheral blood lymphocyte subset reference ranges from the present study with those published previously. Table S3. Distribution and reference ranges of lymphocyte subsets based on age and sex. [file 12865_2020_374_MOESM1_ESM.doc]

Additional file 1

**Table S1.** Reference ranges of peripheral blood lymphocyte subsets in 150 healthy adults.

| **Parameters** | **Mean** | **Median** | **Interquartile ranges** | **Reference ranges *^a)^*** |
| --- | --- | --- | --- | --- |
| **Lymphocyte subsets (%)** | | | | |
| T | 70.97 | 71.63 | 9.65 | 56.99-83.39 |
| CD4^+^ T | 36.59 | 36.46 | 10.25 | 23.78-51.07 |
| CD8^+^ T | 28.01 | 27.11 | 11.08 | 15.00-47.94 |
| B | 10.84 | 10.63 | 5.19 | 5.00-20.06 |
| NK | 16.30 | 15.83 | 9.87 | 4.97-31.09 |
| CD4: CD8 | 1.44 | 1.36 | 0.77 | 0.61-2.80 |
| **Lymphocyte subsets (cells/μl)** | | | | |
| T | 1362 | 1329 | 446 | 832-2093 |
| CD4^+^ T | 693 | 647 | 264 | 360-1127 |
| CD8^+^ T | 534 | 496 | 255 | 249-1057 |
| B | 208 | 193 | 114 | 67-453 |
| NK | 320 | 294 | 242 | 85-775 |

The different lymphocyte subsets (percentages and absolute counts) were gated from CD45^+^ cells and were automatically calculated using BD Multitest software. *^a)^* Reference ranges are defined as 95% of the population.

**Table S2. Comparison of peripheral blood lymphocyte subset reference ranges from the present study with those published previously.**

| **Parameters** | | **Chinese** | | | **Asian** | | | **European** | | | | **North America** | **Middle Eastern (Oman)** | | |
| --- | --- | --- | --- | --- | --- | --- | --- | --- | --- | --- | --- | --- | --- | --- | --- |
|  |  | **Present** | **Beijing** ^[9]^ | **Hong Kong** ^[10]^ | **Singapore**  ^[11]^ | | **Korea** ^[12]^ | **Germany** ^[13]^ | | | | **United States** ^[14]^ | **2008** ^[15]^ | **2013** ^[16]^ | |
| *n* (male/female) | | 150 (75/75) | 151 (100/51) | 273 (150/123) | 232 (104/128) | | 294 (139/155) | 100 (50/50) | | 100  (33/67) | | | 118  (118/0) | 50 (25/25) | |
| Age (years) | |  |  |  |  | |  |  | |  | | |  |  | |
| Mean | | 42.8 | 52 | 36.5 | n.a. | | 47 | n.a. | | 38.1 | | | 25 | n.a. | |
| Median | | 43 | n.a. | 37 | n.a. | | n.a. | 43.4 | | 38 | | | n.a. | 26 | |
| Range | | 20-70 | 19-86 | 17-59 | 16-65 | | 21-80 | 19-85 | | 21-67 | | | 18-51 | 18-57 | |
| Race | | Chinese Han | Chinese | Chinese | Chinese (79.3%), Malay (9.5%), Indian (8.3%) and others(3.9%) | | Korean | n.a. | | n.a. | | | Omani | Omani | |
| T | |  |  |  |  | |  |  | |  | | |  |  | |
| % | 56.98-83.38 | | 43.7-80.5 | 56.09-84.32 | 49.0-80.0 | 45.31-82.81 | | 53-83 | | | 65-88 | | 34-89 | | 57-89 |
| cells/μl | 832-2093 | | 711-2353 | 723-2271 | 796-2679 | 708-2294 | | 780-2240 | | | 983-3572 | | 682.5-3015.6 | | n.a. |
| CD4^+^ T | |  |  |  |  | |  |  | |  | | |  |  | |
| % | 23.77-51.07 | | 22.5-55.1 | 28.06-53.39 | 23.0-48.2 | 25.94-59.73 | | 30-59 | | | 26-62 | | 23-58 | | 31-58 |
| cells/μl | 360-1127 | | 368-1632 | 396-1309 | 401-1451 | 394-1574 | | 490-1640 | | | 491-2000 | | 381.3-1868.4 | | n.a. |
| CD8^+^ T | |  |  |  |  | |  |  | |  | | |  |  | |
| % | 15.00-47.93 | | 11.2-43.1 | 16.37-42.65 | 13.4-41.0 | 11.38-40.51 | | | 10-40 | 14-44 | | | 14-51 | | 19-43 |
| cells/μl | 249-1057 | | 201-931 | 224-1014 | 243-1206 | 188-830 | | | 170-880 | 314-2087 | | | 262.5-1494.3 | | n.a. |
| B | |  |  |  |  | |  |  | |  | | |  |  | |
| % | | 5.00-20.05 | 4.4-21.2 | 7.19-25.85 | 7.0-28.0 | | 3.90-19.70 | 5-21 | | 2-27 | | | 4-28 | 6-23 | |
| cells/μl | | 67-453 | 74-534 | 118-645 | 133-714 | | 57-461 | 80-490 | | 64-800 | | | 348.8-818.4 | n.a. | |
| NK | |  |  |  |  | |  |  | |  | | |  |  | |
| % | | 4.96-31.08 | 3.7-46.1 | 3.66-26.74 | 6.0-37.0 | | 5.11-33.78 | 5-32 | | 2-27 | | | 3-37 | 3-20 | |
| cells/μl | | 85-775 | 63-1013 | 61-607 | 115-1009 | | 91-682 | 80-690 | | 27-693 | | | 71.1-1098.9 | n.a. | |
| CD4: CD8 | | 0.61-2.80 | 0.63-3.49 | 0.71-2.82 | 0.69-2.83 | | 0.77-4.42 | 0.9-5.0 | | 0.6-4.4 | | | n.a. | n.a. | |

n.a., not available.

**Table S3.** Distribution and reference ranges of lymphocyte subsets based on age and sex.

| **Parameters** | **Age (years)** | **Median** | | | ***P*-value** | | **Reference ranges *^a)^*** | |
| --- | --- | --- | --- | --- | --- | --- | --- | --- |
|  |  | **Male** | **Female** | |  |  | **Male** | **Female** |
| **Lymphocyte subsets (%)** | | | | | | | | |
| T | ≤ 40 | 68.71 | 70.00 | 0.155 | | 51.94-82.86 | | 60.18-83.00 |
|  | > 40 | 72.46 | 72.89 |  |  | 51.74-89.88 | | 58.02-84.64 |
| CD4^+^T | ≤ 40 | 30.88 | 36.00 | <0.001 ***^b)^*** | | 22.67-46.00 | | 27.82-49.00 |
|  | > 40 | 37.34 | 39.96 |  |  | 22.86-52.63 | | 24.15-52.95 |
| CD8^+^ T | ≤ 40 | 30.55 | 27.33 | 0.324 | | 15.00-44.07 | | 17.00-43.72 |
|  | > 40 | 25.49 | 25.00 |  |  | 17.30-51.98 | | 12.07-48.33 |
| B | ≤ 40 | 11.22 | 11.00 | 0.428 | | 3.01-17.20 | | 5.00-20.00 |
|  | > 40 | 9.00 | 10.82 |  |  | 3.19-20.59 | | 5.42-22.90 |
| NK | ≤ 40 | 19.02 | 15.65 | 0.018 ***^b)^*** | | 4.85-31.70 | | 5.00-26.00 |
|  | > 40 | 16.00 | 13.00 |  |  | 5.12-37.66 | | 3.02-26.19 |
| CD4:CD8 | ≤ 40 | 1.06 | 1.33 | 0.005 ***^c)^*** | | 0.70-3.02 | | 0.64-2.76 |
|  | > 40 | 1.39 | 1.54 |  |  | 0.46-2.95 | | 0.74-3.97 |
| **Lymphocyte subsets (cells/μl)** | | | | | | | | |
| T | ≤ 40 | 1377 | 1313 | 0.196 | | 758-2267 | | 875-2050 |
|  | > 40 | 1360 | 1247 |  |  | 864-2097 | | 782-2406 |
| CD4^+^ T | ≤ 40 | 645 | 600 | 0.819 | | 283-1145 | | 343-1122 |
|  | > 40 | 678 | 651 |  |  | 348-1283 | | 364-1502 |
| CD8^+^ T | ≤ 40 | 579 | 512 | 0.032 ***^b)^*** | | 309-1055 | | 311-842 |
|  | > 40 | 467 | 455 |  |  | 244-1355 | | 160-1078 |
| B | ≤ 40 | 198 | 198 | 0.805 | | 52-486 | | 92-451 |
|  | > 40 | 166 | 192 |  |  | 63-428 | | 54-404 |
| NK | ≤ 40 | 386 | 282 | 0.002 ***^b)^*** | | 120-1128 | | 118-553 |
|  | > 40 | 302 | 206 |  |  | 75-896 | | 52-702 |

***^a)^*** Reference ranges are defined as 95% of the population. ***^b)^*** Numbers or ***^c)^*** ratio of cell subtypes with significant differences among members of age- and sex-grouped populations. Statistics: Kruskal-Wallis χ^2^ test.
