# Supplementary figures and images for: Characteristics and reference ranges of CD4+T cell subpopulations among healthy adult Han Chinese in Shanxi Province, North China
Source: BMC Immunol. 2020 Aug 3;21:44. doi: 10.1186/s12865-020-00374-9 (PMC7397677; doi:10.1186/s12865-020-00374-9)

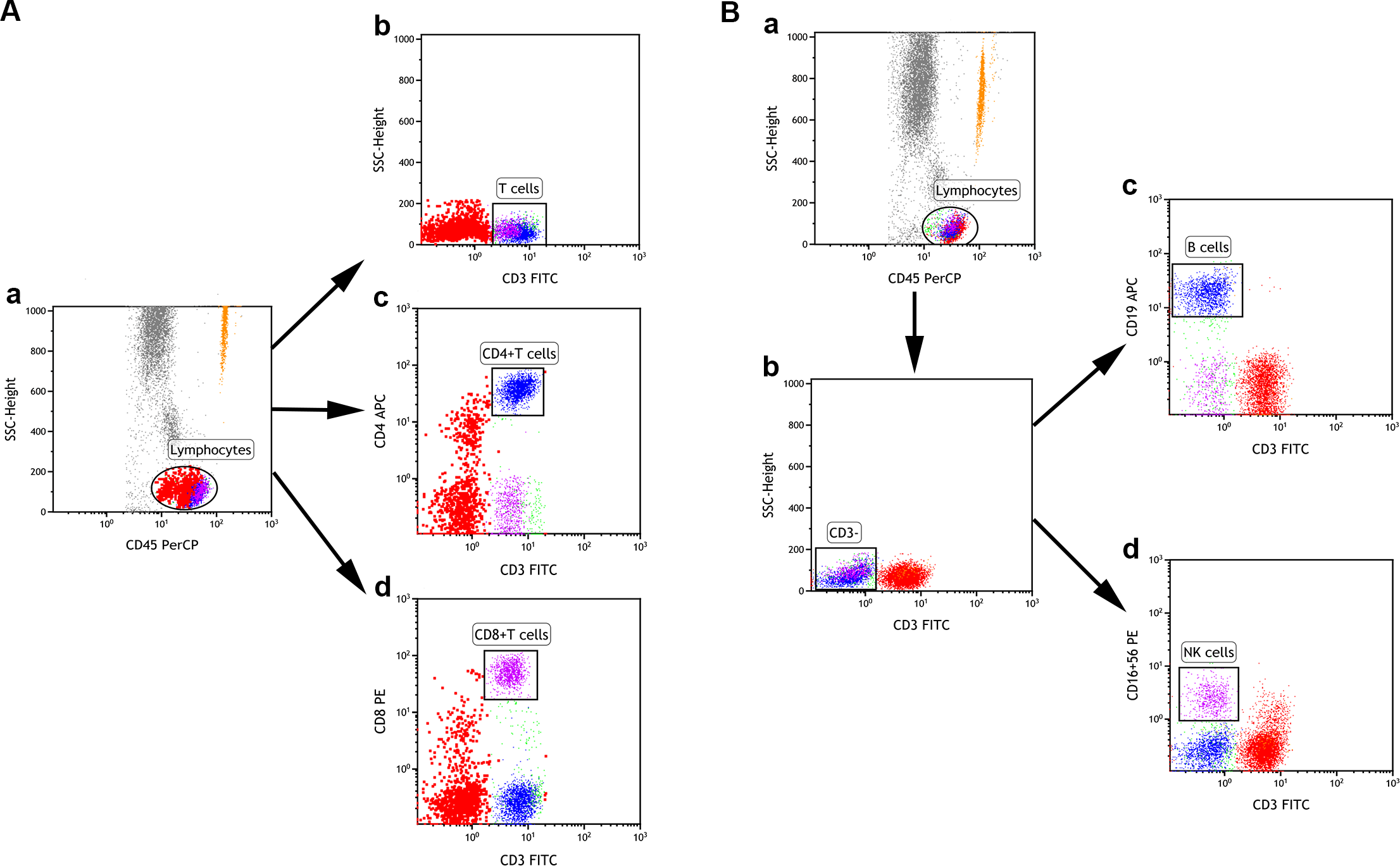

Supplement: Supplementary file 2 — Additional file 2 Figure S1. Analysis of lymphocyte subsets by flow cytometry. A: Gating strategy for T cells, CD4+T cells, CD8+T cells: a expressed as a single parameter of CD45 and scatter; b T cells (CD3+); c CD4+T cells (CD3+CD4+); d CD8+T cells (CD3+CD8+). B: Gating strategy for B cells and NK cells: a expressed as a single parameter of CD45 and scatter; b expressed as a single parameter of CD3− and scatter; c: B cells (CD3−CD19+); d: NK cells (CD3−CD16+CD56+). Figure S2. Analysis of CD4+T subpopulations by flow cytometry. A: Gating strategy for Th1 and Th17 cells: a expressed as a single parameter of CD4 and scatter; b Th1 (CD4+IFN-γ+); c Th17 (CD4+IL-17+). B: Gating strategy for Th2 cells: a expressed as a single parameter of CD4 and scatter; b Th2 (CD4+IL-4+). C: Gating strategy for Treg cells: a expressed as a single parameter of CD4 and scatter; b Treg (CD4+CD25+Foxp3+). [file 12865_2020_374_MOESM2_ESM.zip › Figure S1 .tif]

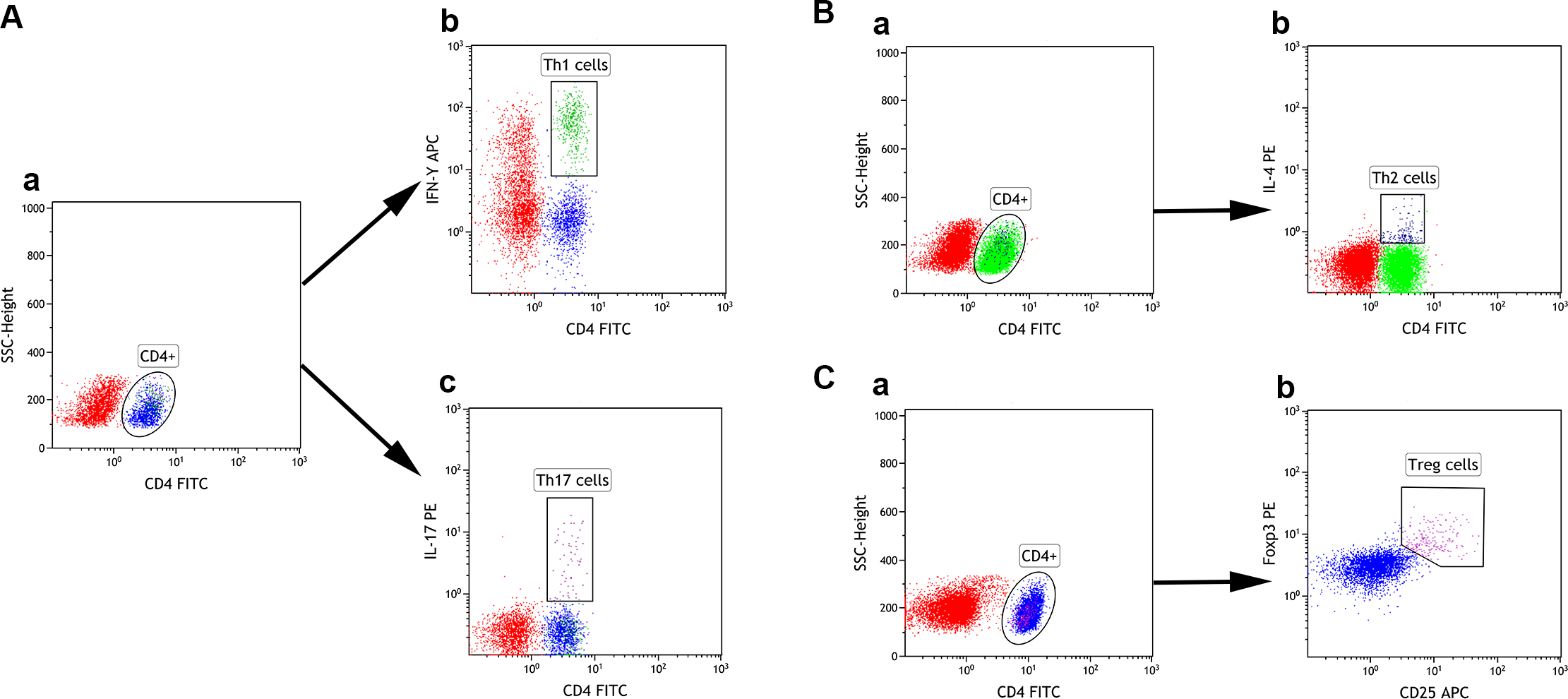

Supplement: Supplementary file 2 — Additional file 2 Figure S1. Analysis of lymphocyte subsets by flow cytometry. A: Gating strategy for T cells, CD4+T cells, CD8+T cells: a expressed as a single parameter of CD45 and scatter; b T cells (CD3+); c CD4+T cells (CD3+CD4+); d CD8+T cells (CD3+CD8+). B: Gating strategy for B cells and NK cells: a expressed as a single parameter of CD45 and scatter; b expressed as a single parameter of CD3− and scatter; c: B cells (CD3−CD19+); d: NK cells (CD3−CD16+CD56+). Figure S2. Analysis of CD4+T subpopulations by flow cytometry. A: Gating strategy for Th1 and Th17 cells: a expressed as a single parameter of CD4 and scatter; b Th1 (CD4+IFN-γ+); c Th17 (CD4+IL-17+). B: Gating strategy for Th2 cells: a expressed as a single parameter of CD4 and scatter; b Th2 (CD4+IL-4+). C: Gating strategy for Treg cells: a expressed as a single parameter of CD4 and scatter; b Treg (CD4+CD25+Foxp3+). [file 12865_2020_374_MOESM2_ESM.zip › Figure S2 .tif]
