# Supplementary material for: Characteristics and reference ranges of CD4+T cell subpopulations among healthy adult Han Chinese in Shanxi Province, North China
Source: BMC Immunol. 2020 Aug 3;21:44. doi: 10.1186/s12865-020-00374-9 (PMC7397677; doi:10.1186/s12865-020-00374-9)
Supplement: Supplementary file 2 — Additional file 2 Figure S1. Analysis of lymphocyte subsets by flow cytometry. A: Gating strategy for T cells, CD4+T cells, CD8+T cells: a expressed as a single parameter of CD45 and scatter; b T cells (CD3+); c CD4+T cells (CD3+CD4+); d CD8+T cells (CD3+CD8+). B: Gating strategy for B cells and NK cells: a expressed as a single parameter of CD45 and scatter; b expressed as a single parameter of CD3− and scatter; c: B cells (CD3−CD19+); d: NK cells (CD3−CD16+CD56+). Figure S2. Analysis of CD4+T subpopulations by flow cytometry. A: Gating strategy for Th1 and Th17 cells: a expressed as a single parameter of CD4 and scatter; b Th1 (CD4+IFN-γ+); c Th17 (CD4+IL-17+). B: Gating strategy for Th2 cells: a expressed as a single parameter of CD4 and scatter; b Th2 (CD4+IL-4+). C: Gating strategy for Treg cells: a expressed as a single parameter of CD4 and scatter; b Treg (CD4+CD25+Foxp3+). [file 12865_2020_374_MOESM2_ESM.zip › Additional_file_2__.doc]

Additional file 2

**Figure S1.** Analysis of lymphocyte subsets by flow cytometry. **A**: Gating strategy for T cells, CD4^+^T cells, CD8^+^T cells: **a** expressed as a single parameter of CD45 and scatter; **b** T cells (CD3^+^); **c** CD4^+^T cells (CD3^+^CD4^+^); **d** CD8^+^T cells (CD3^+^CD8^+^). **B**: Gating strategy for B cells and NK cells: **a** expressed as a single parameter of CD45 and scatter; **b** expressed as a single parameter of CD3^−^ and scatter; **c**: B cells (CD3^−^CD19^+^); **d**: NK cells (CD3^−^CD16^+^CD56^+^).

**Figure S2.** Analysis of CD4^+^T subpopulations by flow cytometry. **A**: Gating strategy for Th1 and Th17 cells: **a** expressed as a single parameter of CD4 and scatter; **b** Th1 (CD4^+^IFN-γ^+^); **c** Th17 (CD4^+^IL-17^+^). **B**: Gating strategy for Th2 cells: **a** expressed as a single parameter of CD4 and scatter; **b** Th2 (CD4^+^IL-4^+^). **C**: Gating strategy for Treg cells: **a** expressed as a single parameter of CD4 and scatter; **b** Treg (CD4^+^CD25^+^Foxp3^+^).
